# Improved detection and localization of insulinoma using small field-of-view diffusion-weighted MRI: A case report

**DOI:** 10.1016/j.radcr.2025.04.059

**Published:** 2025-05-12

**Authors:** Quang Trung Nguyen, Van Diep Pham, Quoc Dat Tran, Quang Long Le, Ngoc Cuong Nguyen, Tuan Linh Le

**Affiliations:** Radiology Center, Hanoi Medical University Hospital, Hanoi, Vietnam

**Keywords:** Insulinoma, CT, MRI, Small field-of-view DWI, Pancreatic neuroendocrine tumor

## Abstract

Insulinoma is a rare but challenging-to-diagnose pancreatic neuroendocrine tumor, often presenting with recurrent spontaneous hypoglycemia. Surgical resection remains the definitive treatment; however, accurate preoperative localization is critical for optimizing surgical outcomes and avoiding unnecessary extensive pancreatic resection. We report a case of a 57-year-old female with recurrent hypoglycemia, whose lesion was undetectable using conventional ultrasound, computed tomography (CT), and standard magnetic resonance imaging (MRI). However, small field-of-view diffusion-weighted imaging (sFOV DWI) successfully identified a 13 × 10 mm pancreatic lesion between the body and tail, with imaging characteristics consistent with a pancreatic neuroendocrine tumor. The patient underwent successful surgical resection, with histopathological confirmation of insulinoma, leading to complete resolution of symptoms postoperatively. This case highlights the utility of sFOV DWI in detecting small pancreatic lesions that may be missed by conventional imaging techniques. Its application in challenging cases could significantly improve diagnostic accuracy and guide surgical planning.

## Introduction

Insulinoma is the most common functional pancreatic islet cell tumor, secreting insulin and causing hypoglycemia, with a wide range of clinical presentations [1]. To diagnose insulinomas, clinicians often rely on multiple imaging modalities, including ultrasound, computed tomography (CT), and magnetic resonance imaging (MRI), each offering distinct advantages in lesion detection [2]. On CT or MRI, the typical appearance of insulinomas is hypervascular lesions with enhancement exceeding that of the normal pancreatic parenchyma during the arterial and capillary phases [2]. However, small insulinomas, particularly those without hypervascular features, pose significant diagnostic challenges for conventional imaging techniques. In this study, we highlight the superior role of sFOV DWI in localizing such lesions, particularly in cases where traditional imaging fails. This advanced imaging technique not only facilitates precise surgical planning but also avoids unnecessary invasive interventions for patients.

## Case report

A 57-year-old female presented with a 3-month history of recurrent episodes of hunger, limb weakness, and profuse sweating occurring multiple times per day. Twenty days before admission, she experienced an early-morning episode of loss of consciousness with unresponsiveness and convulsions. She was brought to a local medical center, where capillary blood glucose measurement revealed severe hypoglycemia (1.0 mmol/L). Her condition improved after intravenous glucose administration.

The patient later sought medical evaluation at Hanoi Medical University Hospital due to recurrent symptoms. Laboratory tests revealed a fasting blood glucose level of 2.4 mmol/L. Additional investigations, including electroencephalography (EEG), electrocardiography (ECG), complete blood count, serum biochemistry, serum electrolyte levels, thyroid ultrasound, abdominal ultrasound, and chest X-ray, showed no abnormalities. She was admitted for further evaluation and management of suspected insulinoma. Additional diagnostic tests were conducted to confirm the diagnosis.

**Fig. 1:**
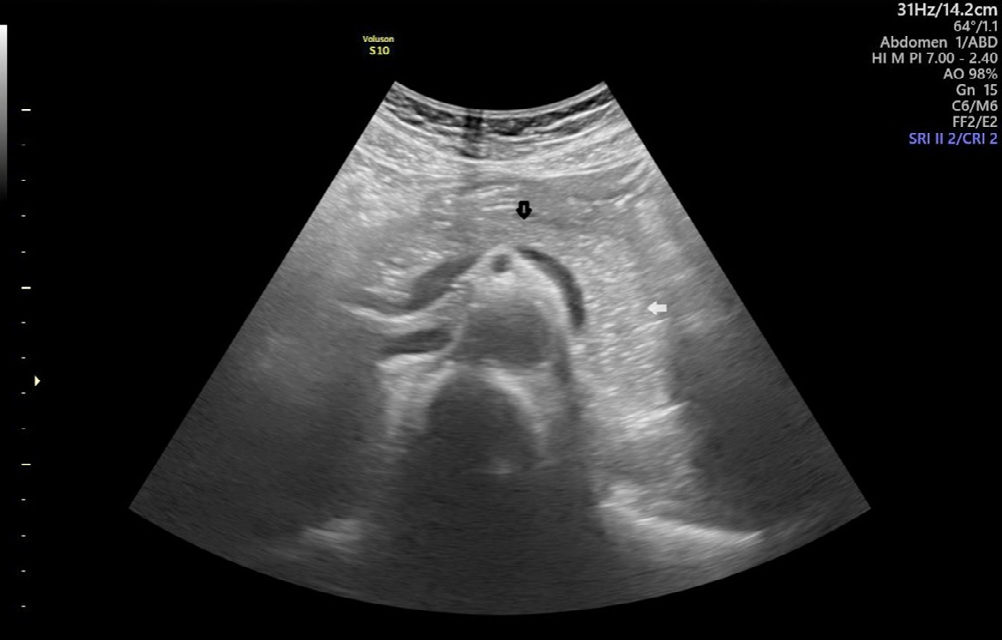
Ultrasound image of the pancreas, with a focused scan on the body and tail region. No hypoechoic or hyperechoic abnormal lesions were detected in the pancreatic body (black arrow) or tail (white arrow).

Abdominal ultrasound (Fig. 1) was performed to evaluate the pancreas, but no abnormalities in the pancreatic parenchyma were detected.

**Fig. 2:**
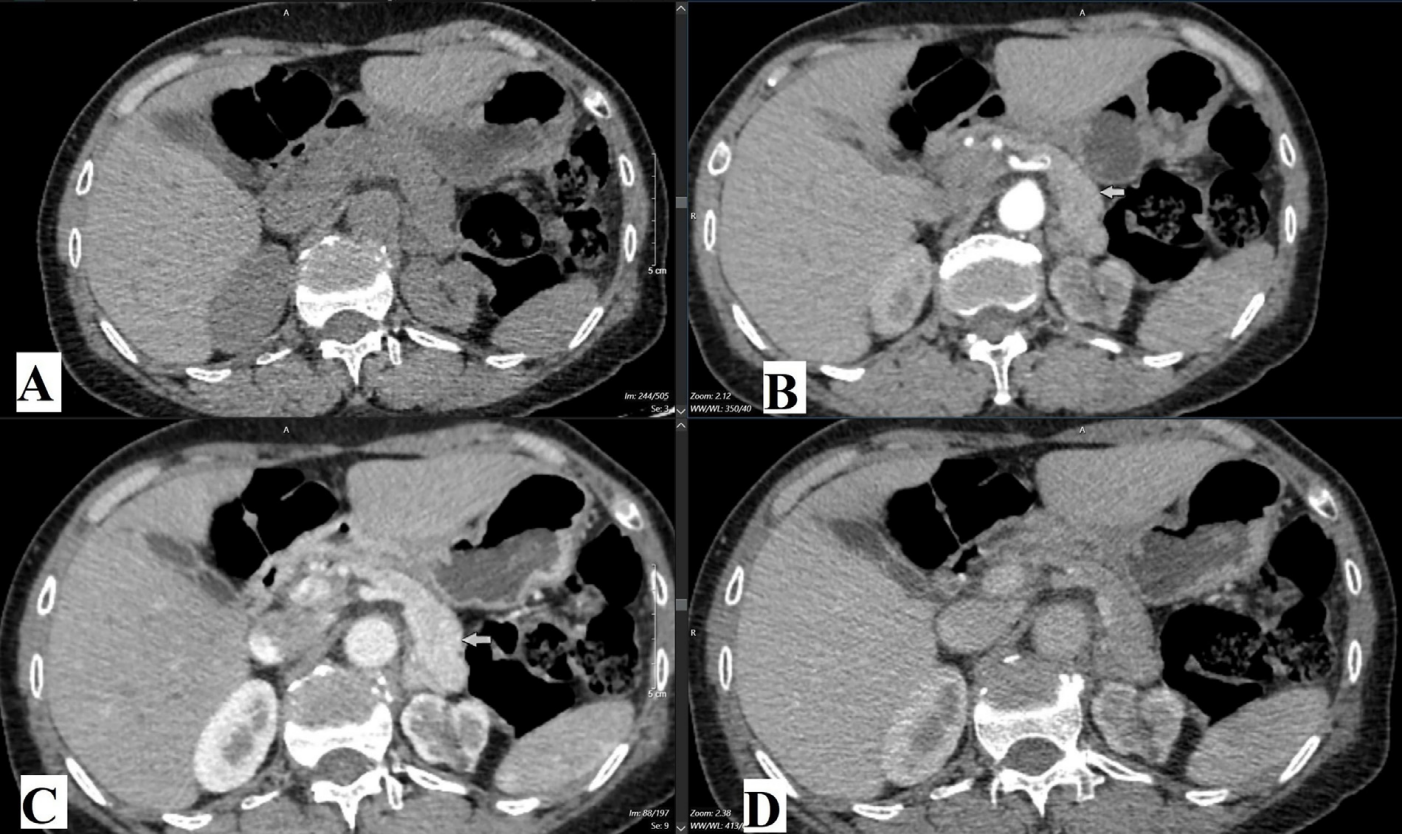
Contrast-enhanced computed tomography (CT) images at different phases: (A) noncontrast phase, (B) arterial phase, (C) portal venous phase, and (D) delayed phase. White arrow in (B) arterial phase, (C) venous phaseindicates the approximate anatomical region of interest (between body and tail of the pancreatic), with no evident contrast enhancement suggestive of a lesion.

Contrast-enhanced abdominal CT (Fig. 2) was performed using a GE Evolution 128-slice CT scanner, with images reconstructed at 1.25 mm intervals at 35, 60, and 150 seconds postcontrast injection. However, no detectable lesion was observed in the pancreatic tail.

**Fig. 3:**
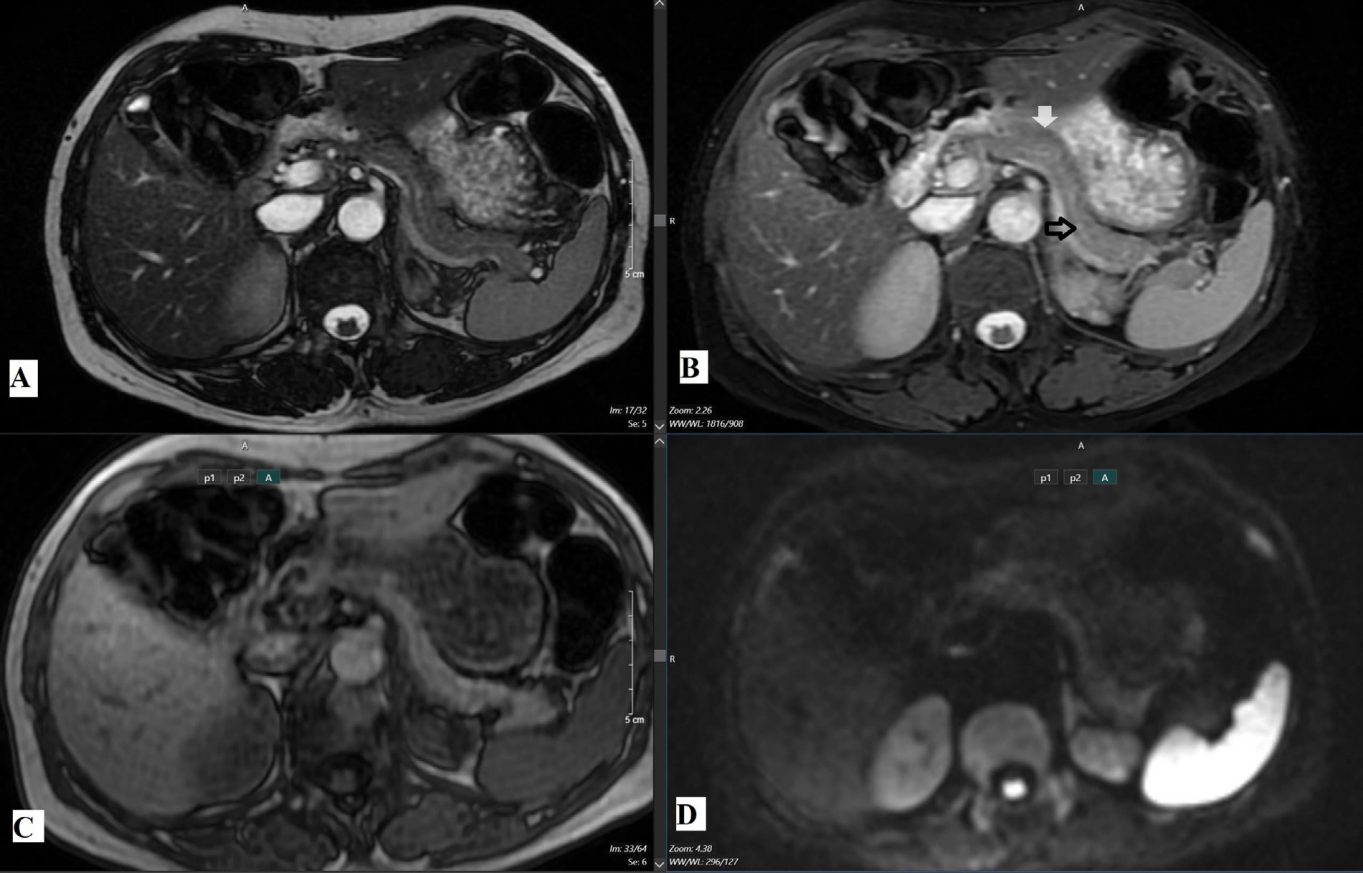
Magnetic resonance imaging (MRI) sequences of the pancreas, assessing the body (white arrow) and tail (black arrow): (A) T2-weighted image (T2W), (B) diffusion-weighted image at b1000 (DWI-b1000), (C) T2-weighted fat-saturated image (T2W fat sat), and (D) T1-weighted in-phase image (T1W in-phase).

**Fig. 4:**
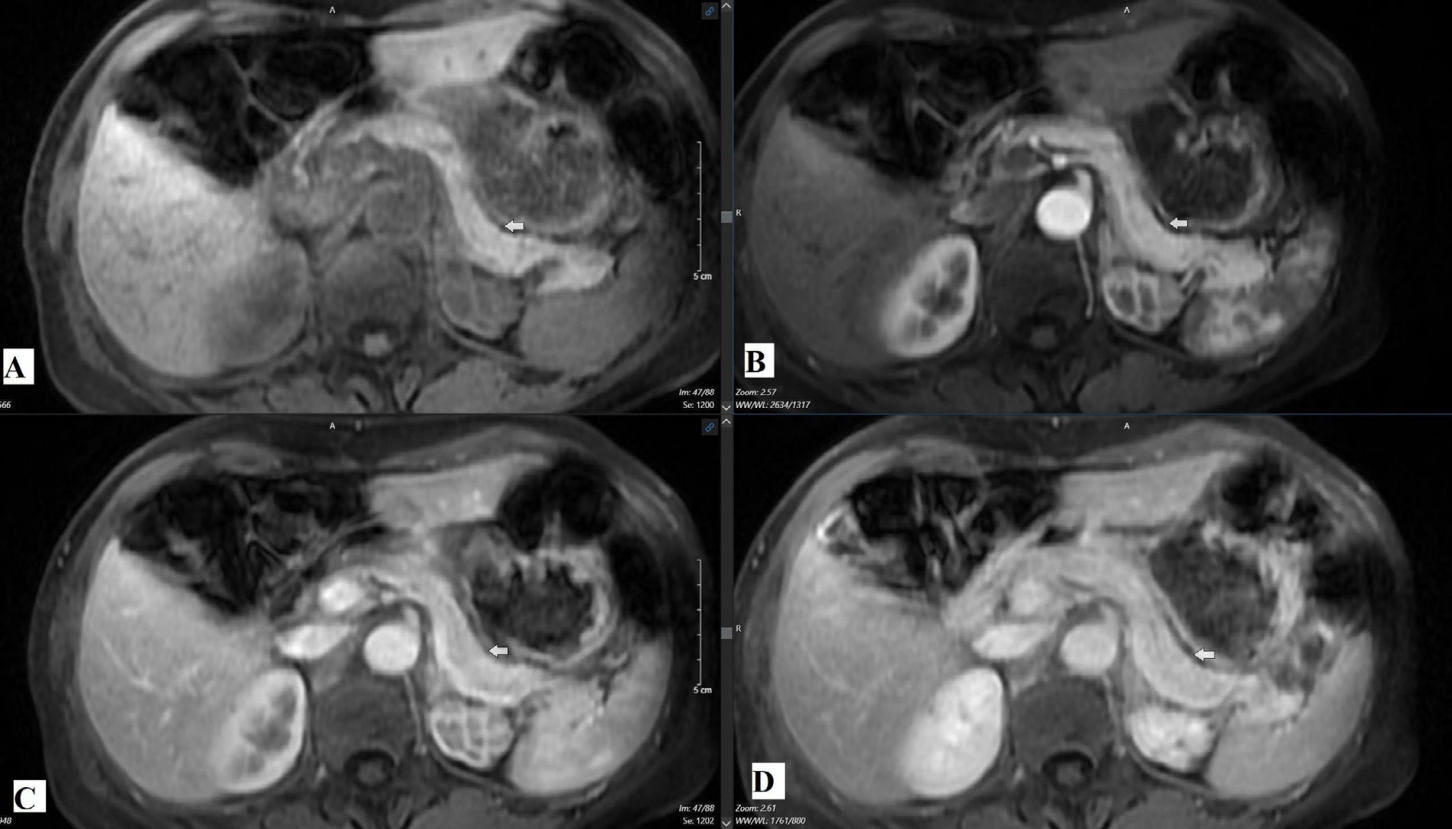
Dynamic contrast-enhanced MRI phases: (A) noncontrast phase, (B) arterial phase, (C) venous phase, and (D) delayed phase. Arrows white in figure A, B, C and D indicate the expected region of the pancreatic tail where no focal lesion was visualized.

MRI (Fig. 3, Fig. 4) was conducted using a 1.5 Tesla GE scanner with standard sequences, including T2-weighted imaging, in-phase and out-phase T1-weighted imaging, diffusion-weighted imaging (DWI), and dynamic contrast-enhanced imaging with gadolinium. Similar to CT, MRI did not reveal any abnormalities in the pancreatic tail region.

**Fig. 5:**
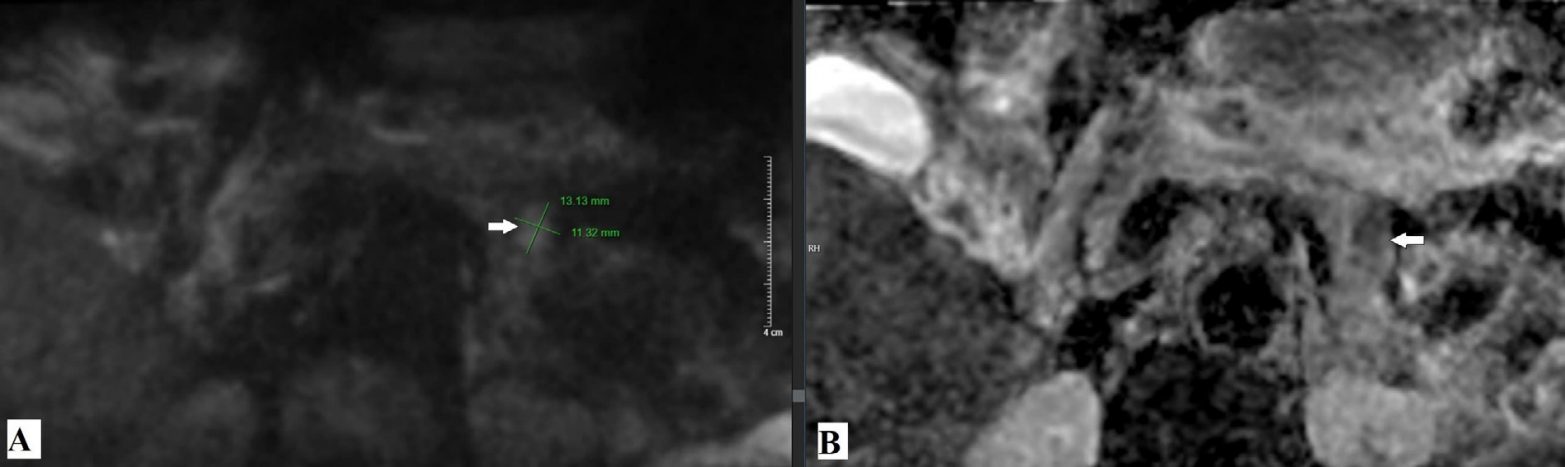
Small field-of-view (sFOV) diffusion-weighted imaging (DWI) sequence with a b-value of 1000 s/mm².(A) The image demonstrates a focal area of restricted diffusion located between the pancreatic body and tail, measuring approximately 13 × 11 mm (white arrow). (B) The corresponding apparent diffusion coefficient (ADC) map shows a hypointense region in the same location (white arrow).

With conventional imaging modalities, including ultrasound, computed tomography (CT), and magnetic resonance imaging (MRI), failing to identify a pancreatic lesion despite the patient's characteristic clinical presentation of insulinoma, the radiologist was tasked with determining whether a pancreatic lesion was present. A small field-of-view diffusion-weighted imaging (sFOV DWI) sequence (Fig. 5), optimized for pancreatic evaluation, was subsequently performed. This revealed a well-defined 13 × 11 mm lesion located between the pancreatic body and tail, demonstrating restricted diffusion and reduced signal intensity on the apparent diffusion coefficient (ADC) map. The imaging parameters included an FOV of 24, slice thickness of 5 mm, matrix size of 150 × 74, and a scan time of 4 minutes.

**Fig. 6:**
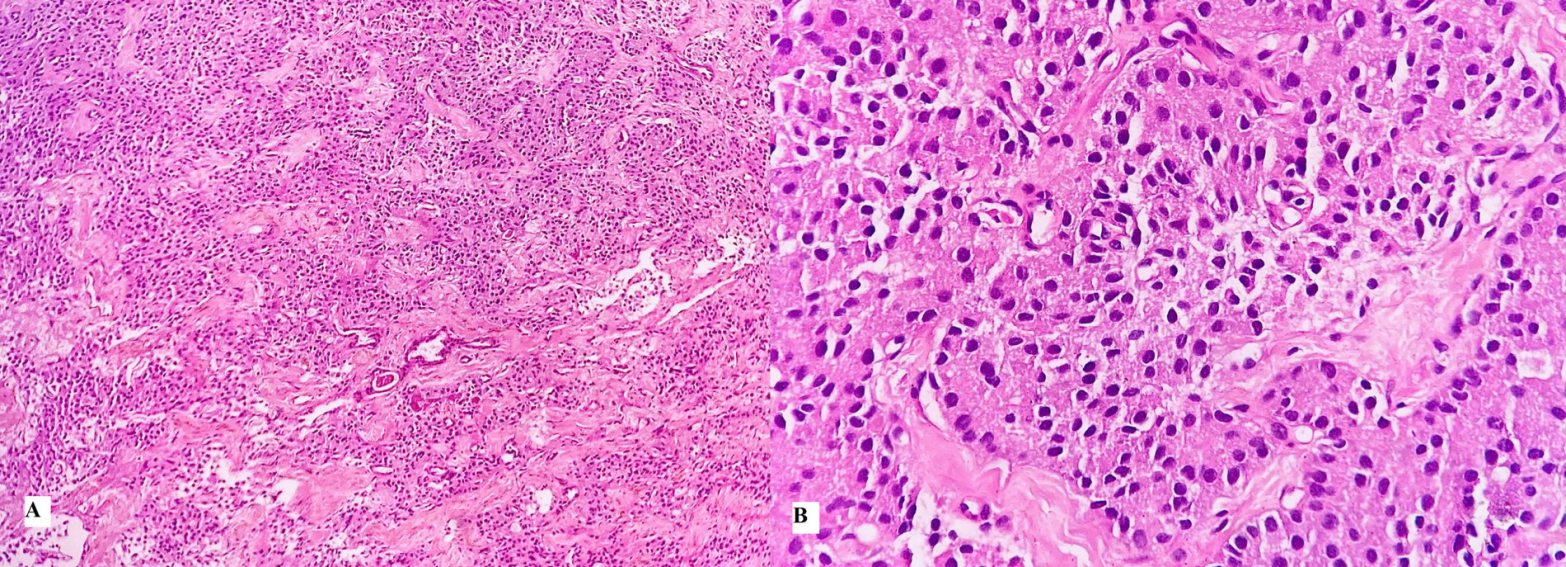
Histopathological features of a Grade 1 pancreatic neuroendocrine tumor (pNET). The tumor cells exhibit uniform, round to oval nuclei arranged in glandular structures, with no mitotic activity or necrosis observed. (A) Hematoxylin and eosin staining, magnification × 100. (B) Hematoxylin and eosin staining, magnification × 400.

The patient underwent successful resection of the pancreatic tail lesion. Histopathology confirmed a grade 1 pancreatic neuroendocrine tumor (Fig. 6). Postoperatively, the patient’s symptoms resolved completely. Follow-up tests at discharge, 3 months, and 6 months showed normal glucose and insulin levels.

## Discussion

Before hospital admission, the patient experienced symptoms of shakiness and weakness in the limbs accompanied by excessive sweating multiple times per day. Notably, the patient had episodes of early-morning unconsciousness, during which they were unresponsive and exhibited seizures. However, normal electroencephalogram (EEG) results ruled out organic neurological damage. Conversely, low blood glucose levels indicated neurological symptoms caused by hypoglycemia beyond the threshold, likely due to a lesion that excessively secretes insulin [3], as the human brain relies solely on glucose as its energy source. Thus, hypoglycemia significantly impacts neurological function, consciousness, and can damage the brain.

Ultrasound is the first-line, noninvasive, and widely accessible imaging modality at all healthcare levels. However, detecting insulinomas with routine preoperative ultrasound is challenging, with a success rate of only 25%-60% of cases [4]. The main difficulty in identifying pancreatic insulinomas lies in the anatomical structure of the pancreas and the small size of the tumor at the time of clinical presentation [5].

Due to its wide field of view, CT remains a key imaging tool for identifying pancreatic neuroendocrine tumors (PNETs). However, its sensitivity in detecting insulinomas varies widely (30%-80%) and depends largely on tumor size and contrast enhancement patterns. Gastrinomas are slightly easier to detect due to their larger size [2,6,7].

On CT imaging, pancreatic neuroendocrine tumors appear as hypervascular lesions with greater enhancement than normal pancreatic parenchyma during the arterial and capillary phases, resulting in higher attenuation [1,2,8]. However, neuroendocrine tumors may also exhibit atypical imaging characteristics, as in this case, potentially leading to false-negative diagnoses.

MRI has been reported to have a sensitivity of 93% and a specificity of 88% [9]. MRI drawbacks include motion artifacts due to breathing, prolonged examination times, and contraindications in patients sensitive to magnetic fields. However, with advancements in diffusion-weighted imaging (DWI), combining conventional pulse sequences with high b-value diffusion sequences shows promise in improving the detection of insulinomas, particularly in patients with small, isointense lesions on standard MRI sequences. This approach eliminates the need for gadolinium contrast agents, avoiding associated contraindications in CT-MRI [10,11].

In this clinical case, despite using both basic and diffusion-weighted pulse sequences during MRI, no lesions were detected between the pancreatic body and tail. This suggests that MRI-based detection of pancreatic lesions may produce false-negative results due to artifacts caused by the pancreas's deep abdominal location.

Diffusion-weighted MRI is highly sensitive for detecting and characterizing both benign and malignant lesions [12]. However, in an organ like the pancreas, motion artifacts and gas interference from adjacent gastrointestinal structures often occur. Using DWI sequences with a small field of view (sFOV) focused on the pancreas reduces such artifacts, increases resolution, and enhances sensitivity for detecting small pancreatic lesions, as demonstrated in previous studies [13]. Specifically, sFOV imaging has been shown to improve image quality, including anatomical structure visualization, contrast-to-noise ratio (CNR), and lesion clarity. It also minimizes artifacts such as shadowing, sensitivity to motion, and jagged noise compared to conventional diffusion techniques using a large FOV [13,14].

Endoscopic ultrasound (EUS) is considered the most sensitive modality for insulinoma detection, with reported sensitivity rates of 93% [15]. However, it is an invasive technique that requires specialized expertise, and its accuracy may be operator-dependent. Gallium-68 DOTATATE PET/CT has been widely used for neuroendocrine tumor detection, including insulinomas, with high sensitivity. Haug et al. [16] demonstrated its efficacy in detecting neuroendocrine tumor recurrence (sensitivity, 90%), emphasizing its role in both primary diagnosis and follow-up imaging. However, its limited availability and high cost remain challenges for widespread clinical use

In comparison, small field-of-view diffusion-weighted imaging (sFOV DWI) provides a noninvasive alternative with enhanced lesion conspicuity and improved spatial resolution. Recent studies have demonstrated that sFOV DWI has a significantly higher detection rate for insulinomas compared to conventional full-field DWI (92.4% vs 77.3%, *P* = .013) [17]. Its ability to highlight small pancreatic lesions with reduced motion artifacts makes it a valuable adjunct to standard MRI, especially in cases where traditional imaging fails to identify insulinomas.

**Table:** 

Imaging Modality	Sensitivity (%)	Advantages	Limitations
Ultrasound (US)	25-60	Noninvasive, widely available	Operator-dependent, low sensitivity for small tumors
Dynamic CT	30-80	Widely available, fast	Low sensitivity for small/nonhypervascular lesions
Conventional MRI	88-93	Noninvasive, good contrast resolution	Motion artifacts, may miss small/isointense tumors
Endoscopic Ultrasound (EUS)	85-94	High sensitivity for small tumors	Invasive, operator-dependent
PET-CT (Ga-DOTATATE)	90	Whole-body assessment, high sensitivity for NETs	Expensive, limited availability
sFOV DWI (MRI)	92.4	Noninvasive, better for small lesions	Longer scan time, requires specialized MRI settings

Given these findings, sFOV DWI should be considered an essential imaging tool in cases where conventional CT or MRI fails to detect insulinomas. While EUS and PET-CT remain highly sensitive, their limitations in invasiveness and cost suggest that sFOV DWI may serve as an optimal noninvasive alternative for insulinoma localization.

It is important to note that patient-related factors, particularly body habitus and positioning, can influence the diagnostic performance of small field-of-view diffusion-weighted imaging (sFOV DWI). In obese patients, increased soft tissue thickness may degrade image quality by reducing the signal-to-noise ratio (SNR) and increasing motion-related artifacts, thereby limiting lesion conspicuity and diagnostic confidence [14,17]. Furthermore, respiratory motion and gastrointestinal peristalsis can exacerbate these artifacts, especially when imaging deep abdominal structures such as the pancreas [18].

However, sFOV DWI offers several technical advantages that partially mitigate these limitations. By focusing on a targeted anatomical region, this technique reduces phase-encoding errors, enhances spatial resolution, and suppresses background noise, making it more robust against common artifacts compared to conventional full FOV DWI [13,14,17]. Although promising, further studies with larger and more diverse patient cohorts are warranted to systematically assess the impact of body habitus and patient positioning on sFOV DWI accuracy and reproducibility. In this clinical case, the use of sFOV DWI confirmed the value of this technique in providing critical information for identifying a lesion between the pancreatic body and tail, which was undetectable by conventional CT and MRI. The diagnosis was validated by the surgeon, with histopathological results confirming the lesion, and the patient’s clinical condition showed significant improvement following surgical removal of the pancreatic lesion.

Clinical Significance and Limitations of sFOV DWI: The sFOV DWI technique not only improves the ability to detect small insulinoma lesions but also provides additional information regarding lesion characteristics, facilitating the differentiation between benign and malignant tumors. This plays a crucial role in optimizing treatment strategies and patient management. However, several limitations need to be taken into account:

1. Cost and technical requirements: MRI systems must be equipped with the appropriate software and hardware to support sFOV DWI, which may not be readily available in all healthcare facilities.
2. Prolonged scanning time: sFOV DWI generally requires more time compared to conventional imaging methods, which can pose challenges for unstable patients or pediatric populations.
3. Not a complete substitute for other modalities: Although highly effective, sFOV DWI cannot entirely replace ultrasound, CT, or conventional MRI. Instead, it should be utilized as an adjunctive tool in challenging diagnostic cases.

## Recommendations

Based on these findings, the application of sFOV DWI should be considered in cases of suspected insulinoma where lesions are not detected by ultrasound, CT, or conventional MRI. This approach could significantly enhance diagnostic and therapeutic effectiveness, particularly in large medical centers or specialized hospitals.

## Conclusion

Insulinoma is the most common islet cell tumor of the pancreas. Diagnostic modalities such as ultrasound, computed tomography (CT), and magnetic resonance imaging (MRI) generally provide substantial information about the lesion. However, in certain cases with clear clinical symptoms but negative findings on conventional imaging, the use of diffusion-weighted imaging (DWI) with a small field of view (sFOV) localized to the pancreas can serve as a valuable solution to confirm the diagnosis. This approach helps to avoid missing lesions, which could lead to ineffective treatment strategies. A comprehensive diagnostic approach, combining clinical symptoms, laboratory markers, imaging modalities, and histopathological confirmation, is essential for accurate identification and management of insulinomas. determining their number, assessing their relationship with adjacent structures, and pinpointing their exact location. Future studies should further validate its clinical utility in larger patient cohorts

## Patient consent

Informed consent for patient information to be published in this article was obtained.
